# Risk of obstetric anal sphincter injury increases with maternal age irrespective of parity: a population-based register study

**DOI:** 10.1186/s12884-017-1473-7

**Published:** 2017-09-15

**Authors:** Ulla Waldenström, Cecilia Ekéus

**Affiliations:** 1 Department of Women’s and Children’s Health, Division of Reproductive Health, Karolinska Institutet, Retzius väg 13 a-b, 171 77 Stockholm, Sweden; 2 Bastugatan 42, 118 25, Stockholm, Sweden

**Keywords:** Obstetric anal sphincter injury, OASI, Maternal age, Parity

## Abstract

**Background:**

Obstetric anal sphincter injury (OASI) is a rare but serious outcome of vaginal birth. Based on concerns about the increasing number of women who commence childbearing later than previous generation, this study aimed at investigating age-related risk of OASI in women of different parity.

**Methods:**

A population-based register study including 959,559 live singleton vaginal births recorded in the Swedish Medical Birth Register 1999 to 2011. In each parity group risks of OASI at age 25–29 years, 30–34 years, and ≥35 years compared with age < 25 years were investigated by logistic regression analyses, adjusted for year of birth, education, region of birth, smoking, Body Mass Index, infant birthweight and fetal presentation; and in parous women, history of OASI and cesarean section. Additional analyses also adjusted for mediating factors, such as epidural analgesia, episiotomy, and instrumental delivery, and maternal age-related morbidity.

**Results:**

Rates of OASI were 6.6%, 2.3% and 0.9% in first, second and third births respectively. Age-related risk increased from 25-29 years in first births (Adjusted OR 1.66; 95% CI 1.59–1.72) and second births (Adjusted OR 1.78; 95% CI 1.58–2.01), and from 30-34 years in third births (Adjusted OR 1.60; 95% CI 1.00–2.56). In all parity groups the risk was doubled at age ≥ 35 years, compared with the respective reference group of women under 25 years. Adding mediating factors and maternal age-related morbidity only marginally reduced these risk estimates.

**Conclusion:**

Maternal age is an independent risk factor for OASI in first, second and third births. Although age-related risks by parity are relatively similar, more nulliparous than parous women will be exposed to OASI due to the higher baseline rate.

**Electronic supplementary material:**

The online version of this article (10.1186/s12884-017-1473-7) contains supplementary material, which is available to authorized users.

## Background

Obstetric anal sphincter injury (OASI), which refers to perineal tears that involve the external and/or internal anal sphincter, is a well-known complication of vaginal birth. Three factors have significantly contributed to the increased attention paid to OASI by clinicians and researchers: the severe consequences in terms of anal incontinence [1–3]; the increasing incidence in many countries, such as the UK [4, 5], Canada [6], Sweden [7], Norway [8] and Finland [9]; and the variation in the reported rates of OASI from less than 2% [10] to 27% in primiparous and 8.5% in multiparous women when assessed by endoanal ultrasonography [11].

In order to understand and reduce the incidence of OASI extensive research has focused on identification of risk factors, many of which are directly or indirectly associated with one another. To simplify, the risk factors can be classified into three clusters related to the infant, the medical procedures and maternal characteristics. An important infant risk factor is high birthweight [4, 7, 9, 12–14], but also large head circumference [8, 15, 16], occiput posterior presentation [7, 15, 16], shoulder dystocia [17], and a long second stage [13, 15, 16, 18, 19]. Procedural risk factors are instrumental vaginal birth [4, 7, 8, 13, 14, 16, 18], and midline episiotomy [7, 20]. Also, birth position [15, 19] and preventive measures applied to the perineum may affect the risk of OASI [21–24], whereas findings regarding the association between OASI and epidural pain relief are inconclusive [7, 12, 15, 16, 18, 19]. Important maternal risk factors are nulliparity [8, 12, 14, 15], and in parous women cesarean section in the previous birth [8, 12, 18], or a previous vaginal birth with OASI [13]. Asian [4, 7, 8, 12] and African [4, 7, 8] ethnic background and advanced maternal age [25–27] are associated with OASI, and age-related morbidity such as diabetes [8, 12] and gestational diabetes [8] have been reported.

The background for the present study was the need to explore in more depth the association between advanced maternal age and the risk of OASI, based on concerns about the increasing number of women who commence childbearing later than previously. Although epidemiological studies cannot ascertain the causes of OASI, they may provide clues about the mechanisms involved. Biological ageing has been the focus of many studies of adverse pregnancy outcomes [28, 29], but less so in studies of OASI. Maternal age has often been treated as a confounding variable and not as the exposure of interest. Comparison groups have been defined differently, resulting in different conclusions. When maternal age was dichotomized, for instance by comparing women younger and older than 35 years, no associations with OASI were found [15, 19, 25], whereas a continuous increase of OASI by maternal age was reported when dividing age into several categories, with the exception of the oldest group of 40 years and over where findings were inconclusive [4, 8, 12–14, 18, 26]. Most studies have either included only primiparous women or all parous women combined. We found no study investigating the association between advanced maternal age in women of different parities. Considering maternal age and parity as two interrelated risk factors that may affect OASI in different ways, it is important to study their combined effect.

The principal aim of the present study was to investigate associations between advanced maternal age and risks of OASI in women’s first, second, and third births in a large population-based cohort. Associations between OASI and other factors, which were regarded as confounding or mediating factors, are also presented.

## Methods

The study was based on data from the Swedish Medical Birth Register (MBR), which includes more than 98% of all births in Sweden, and is validated annually against the National Population Register, using the mother’s and infant’s unique personal identification numbers [30, 31]. Starting at the first antenatal visit, information is prospectively collected during pregnancy and delivery, using standardized records. We included live singleton vaginal births at term (≥37 weeks of gestation), recorded in the MBR from 1999 to 2011. Consequently, a majority of women contributed with information about all their births, some only provided information about their last birth at the beginning of the study period (1999), and others only provided information about their first birth at the end of the period (2011).

From 1999 to 2011, the total number of births in Sweden was 1,301,134. We excluded parity 4 and above (6.0%), multiple births (2.9%), stillbirths (0.3%), preterm births (<37 weeks: 6.2%), cesarean sections (17.2%), and pregnancies without maternal identification number (0.01%), leaving 956,559 pregnancies in the final sample.

The outcome variable was obstetric anal sphincter injury (OASI). Cases were reported by the midwife or the obstetrician using a checkbox in the case notes and/or the notified ICD-10 code (International Classification of Diseases, version 10) for sphincter injury: O702, O702C, O702D, O702X (grade III, which involves the anal sphincter complex) and O703 (grade IV, which extends to the mucosa).

The exposure variable of interest was maternal age when giving birth to the first, second and third infant. In each parity group, maternal age younger than 25 years was used as the reference, and compared with 25–29 years, 30–34 years, and 35 years and over. The rationale for choosing the youngest women as the reference was the assumption that young age would be the most optimal in terms of the elasticity of perineal tissue, and that many studies have reported the lowest OASI rates in the youngest age group [4, 8, 12, 14, 26].

The principal analyses were adjusted for possible confounding factors, including year of birth, education, region of birth, smoking, BMI, infant birthweight and fetal presentation, and in parous women, also history of OASI and history of cesarean section in the previous birth. Information about smoking (dichotomized as daily smoking vs. non-daily smoking), and maternal height and weight were recorded at the first antenatal visit (commonly at 8–12 weeks of gestation). Body Mass Index (BMI) was calculated (weight in kilograms/height in square metres) and categorized according to the World Health Organization as: underweight (BMI <18.5 kg/m^2^), normal weight (BMI 18.5–24.9 kg/m^2^), overweight (BMI 25.0–29.9 kg/m^2^), and obese (BMI ≥30.0 kg/m^2^). Infant birthweight and fetal presentation were recorded shortly after the birth. The Swedish Register of Total Population provided information about the mother’s country of birth (categorized as Sweden, Asia, Africa and Other). Level of education (Elementary school or less, High school, and College or university) was obtained by linkage to the Swedish Education Register. History of OASI and cesarean section was based on information about these outcomes from the previous birth in the dataset.

Additional analyses also included potentially mediating factors, such as epidural analgesia, episiotomy and instrumental vaginal birth (vacuum extraction and forceps), and maternal age-related morbidity (diabetes, gestational diabetes, pregestational hypertension, preeclampsia). Data on maternal diseases were retrieved from the MBR: pregestational diabetes (insulin-dependent or noninsulin-dependent; ICD-9 codes 250 and 648A; ICD-10 codes E10-E14 and O240-O243), gestational diabetes (ICD-9 code 648 W; ICD-10 code O244), pregestational hypertension (self-reported by check box at first antenatal visit or by ICD-9 codes 401–405, 642C and 642H; or by ICD-10 codes 110–115, O10 and O11) and preeclampsia (including eclampsia; ICD-9 codes 642E-642G; ICD-10 codes O14 and O15).

Pregnancies of nulliparous women (1st births), para 1 (2nd births), and para 2 (3rd births) were analyzed separately. Rates of OASI were calculated for each age category and parity group, and also for each of the potentially confounding and mediating factors. The associations between maternal age and OASI were investigated in logistic regression analyses. First, analyses were adjusted for year of birth (Model 1). The principal analyses in Model 2 were also adjusted for mother’s education, region of birth, smoking, BMI, infant birthweight and fetal presentation; and in parous women, also for OASI and cesarean section in the previous pregnancy. Finally, the potentially mediating factors and age-related morbidity were added (Model 3).

In the total sample, missing data were low except for BMI (11.3%) and history of previous OASI and cesarean section (11.1% in second births and 11.7% in third births) (Table 1). Missing data on BMI (maternal weight and height) was substantially explained by the time-point when this information was included in the Medical Birth Register. Missing data on history of previous OASI and cesarean section were related to how the study sample was defined; with information missing in parous women who had their previous birth prior to the onset of the data collection in 1999. We therefore assessed that the missing values in these three variables were likely to be nearly at random (MAR) and thus meeting the criteria for multiple imputation [32]. Imputations were conducted in SPSS Version 22, in three separate datasets including first, second and third births respectively. The SPSS “Automatic” imputation method was used (Type of imputation model: Logistic Regression), and 15 imputations were performed. The imputed variable in the dataset of first births was BMI, in the dataset of second births, BMI and history of OASI and cesarean section in the first birth, and in the dataset of third births, BMI and history of OASI and cesarean section in the second birth. The variables used in the imputation procedure included the outcome variable (OASI), and all the exposure variables listed in Table 1 (except parity). A comparison between the principal findings in Model 2 (based on imputation of missing data on BMI, and history of OASI and cesarean section) and corresponding findings based on complete case analyses are presented in a (Additional file 1: Table S1).

**Table 1:** Variables analyzed, and rates of obstetric anal sphincter injury (OASI) in each variable category: singleton, live, term, vaginal 1st, 2nd and 3rd births, Sweden 1999–2011

Variables	1st births			2nd births			3rd births		
	Prevalence of variables		Rates of OASI	Prevalence of variables		Rates of OASI	Prevalence of variables		Rates of OASI
	n (436670)	%	(%)	n (379787)	%	(%)	n (140102)	%	(%)
Maternal age (years)									
< 25	111,828	25.6	4.1	38,677	10.2	1.0	5232	3.7	0.4
25–29	163,471	37.4	6.9	117,498	30.9	2.0	29,102	20.8	0.6
30–34	121,833	27.9	8.0	153,263	40.4	2.7	57,036	40.7	0.8
≥ 35	39,538	9.1	7.8	70,349	18.5	3.0	48,732	34.8	1.1
Year of birth									
1999–2004	185,654	42.5	7.0	161,407	42.5	2.5	62,560	44.7	1.0
2005–2011	251,016	57.5	6.3	218,380	57.5	2.2	77,542	55.3	0.8
Education									
Elementary: ≤ 9 years	43,820	10.0	4.2	35,170	9.3	1.6	19,189	13.7	0.7
High school	175,631	40.2	6.0	163,317	43.0	2.2	63,376	45.2	0.8
College or university	205,730	47.1	7.6	174,711	46.0	2.6	54,505	38.9	1.0
Missing	11,489	2.6		6589	1.7		3032	2.2	
Country/region of birth									
Sweden	357,730	81.9	6.6	309,967	81.6	2.4	107,752	76.9	0.9
Asia	33,177	7.6	7.4	28,907	7.6	2.3	14,270	10.2	0.9
Africa	7163	1.6	9.0	7171	1.9	3.1	4807	3.4	1.2
Other	38,600	8.8	5.6	33,742	8.9	1.9	13,273	9.5	0.7
Smoking in early pregnancy									
No	381,092	87.3	6.8	335,534	88.3	2.4	119,673	85.4	0.9
Yes	33,734	7.7	3.9	25,689	6.8	1.4	13,573	9.7	0.6
Missing	21,844	5.0		18,564	4.9		6856	4.9	
BMI									
Low: <18.5	12,152	2.8	6.4	7824	2.1	2.1	2015	1.4	0.4
Normal weight: 18.5–24.9	260,128	59.6	6.5	210,403	55.4	2.2	71,198	50.8	0.9
Overweight: 25–29.9	83,185	19.0	6.8	84,411	22.2	2.5	34,887	24.9	0.9
Obese: ≥ 30	31,333	7.2	6.2	35,013	9.2	2.6	15,749	11.2	0.8
Missing	49,872	11.4		42,136	11.1		16,253	11.6	
Infant birthweight (g)									
< 3000	52,966	12.1	2.7	26,046	6.9	0.9	9447	6.7	0.3
3000–3499	163,269	37.4	4.5	111,404	29.3	1.3	38,161	27.2	0.4
3500–3999	157,192	36.0	7.5	150,736	39.7	2.3	54,542	38.9	0.8
4000–4499	53,342	12.2	11.9	73,289	19.3	3.7	29,503	21.1	1.3
≥ 4500	8818	2.0	18.0	17,538	4.6	5.9	8168	5.8	2.6
Missing	1083	0.2		774	0.2		281	0.2	
Presentation									
Occiput anterior	407,784	93.4	6.4	354,776	93.4	2.3	130,554	93.2	0.8
Occiput posterior	14,355	3.3	10.8	12,375	3.3	4.0	4645	3.3	1.5
Breech	1405	0.3	4.3	978	0.3	1.6	327	0.2	0.6
Other	13,126	3.0	6.9	11,658	3.1	2.6	4576	3.3	1.3
Diabetes									
No	435,364	99.7	6.6	378,146	99.6	2.3	139,359	99.5	0.9
Yes	1306	0.3	8.7	1641	0.4	5.0	743	0.5	1.5
Gestational diabetes									
No	433,683	99.3	6.6	376,826	99.2	2.3	138,580	98.9	0.9
Yes	2987	0.7	8.1	2961	0.8	3.3	1522	1.1	1.0
Pregestational hypertension									
No	434,666	99.5	6.6	377,874	99.5	2.3	139,239	99.4	0.9
Yes	2004	0.5	7.0	1913	0.5	2.1	863	0.6	0.9
Preeclampsia									
No	423,794	97.1	6.6	375,404	98.8	2.3	138,380	98.8	0.9
Yes	12,876	2.9	6.2	4383	1.2	2.3	1722	1.2	0.9
Epidural analgesia									
No	234,246	53.6	6.1	308,153	81.1	2.0	121,796	86.9	0.8
Yes	202,424	46.4	7.1	71,634	18.9	3.6	18,306	13.1	1.1
Episiotomy									
No	376,486	86.2	6.2	363,439	95.7	2.2	137,265	98.0	0.8
Yes	60,184	13.8	8.6	16,348	4.3	5.6	2837	2.0	2.5
Vacum extraction or forceps									
No	362,249	83.0	5.0	364,529	96.0	2.0	136,860	97.7	0.8
Yes	74,421	17.0	14.4	15,258	4.0	10.6	3242	2.3	4.3
Second births only									
History of OASI in 1st pregnancy									
No				320,913	84.5	2.0			
Yes				16,531	4.4	7.3			
Missing				42,343	11.1				
History of cesarean section 1st pregnancy									
No				312,869	82.4	1.6			
Yes				24,575	6.5	10.3			
Missing				42,343	11.1				
Third births only									
History of OASI in 2nd pregnancy									
No							121,980	87.1	0.7
Yes							1688	1.2	7.6
Missing							16,434	11.7	
History of cesarean section 2nd pregnancy									
No							121,305	86.6	0.7
Yes							2363	1.7	2.4
Missing							16,434	11.7	

## Results

The rates of OASI in live singleton vaginal births at term were 6.6% in first births, 2.3% in second births, and 0.9% in third births, and increased by maternal age in all parity groups (Table 1). The one exception was in the oldest nulliparae who had about the same rate of OASI as the previous age group of 30–34 years. The incidence of OASI decreased from the first to the second half of the observation period. Table 1 also shows differences in maternal characteristics between the parity groups, which is most obvious in third births where low education, being born in Africa or Asia, smoking, and overweight/obesity were more common than in first and second births. These characteristics were most pronounced in women who had their third birth at young age, that is in the reference group of 25 years and younger (elementary school or less 55%; not born in Sweden 45%; smoking 22%, overweight/obesity 44%). The mediating factors epidural analgesia, episiotomy and instrumental vaginal delivery also differed significantly between the parity groups, with the highest rates in first births and the lowest in third births.

**Fig. 1:**
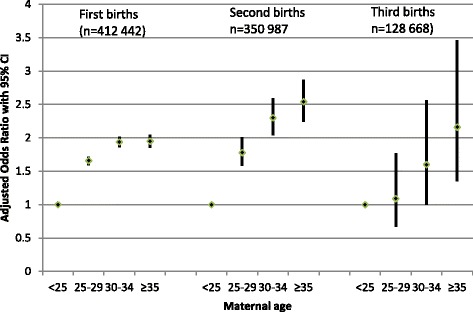
Risk of OASI by maternal age in first, second and third births. Adjusted Odds Ratio with 95% Confidence Interval (CI): Reference 1= < 25 years. Logistic regression analyses adjusted for year of birth, education, country/region of birth, smoking, BMI, infant birthweight, fetal presentation, and in second and third births history of OASI and history of cesarean section in the previous birth

The risk of OASI, expressed as adjusted Odds Ratios, increased almost continuously by maternal age, from the youngest to the oldest, irrespective of parity (Table 2: Model 2). Exceptions were the oldest women (≥35 years) with a first birth, who were at similar risk as the 30–34 year olds, and the 25–29 year olds with a third birth who were at the same risk as the younger reference group. The principal findings in Table 2 (Model 2) are also illustrated in Fig. 1. The associations between maternal age and OASI were very similar to those obtained when data were analysed by complete cases (Additional file 1: Table S1: Model 2 analysed by imputed and complete cases).

**Table 2:** Risk of OASI in first, second and third live, singleton, vaginal births at term

Variables		1st births^1^			2nd births^2^			3rd births^2^	
	Model 1	Model 2	Model 3	Model 1	Model 2	Model 3	Model 1	Model 2	Model 3
	*n* = 436,670	(*n* = 412,442)	(*n* = 412,442)	*n* = 379,787	(*n* = 350,986)	(*n* = 350,982)	(*n* = 140,102)	(*n* = 128,668)	(*n* = 128,668)
	Adj OR (95% CI)	Adj OR (95% CI)	Adj OR (95% CI)	Adj OR (95% CI)	Adj OR (95% CI)	Adj OR (95% CI)	Adj OR (95% CI)	Adj OR (95% CI)	Adj OR (95% CI)
Maternal age (years)									
< 25	1	1	1	1	1	1	1	1	1
25–29	1.75 (1.69–1.82)	1.66 (1.59–1.72)	1.56 (1.50–1.62)	2.05 (1.83–2.29)	1.78 (1.58–2.01)	1.73 (1.53–1.95)	1.29 (0.83–1.99)	1.09 (0.67–1.77)	1.07 (0.66–1.73)
30–34	2.05 (1.98–2.13)	1.94 (1.86–2.02)	1.74 (1.67–1.81)	2.84 (2.55–3.16)	2.30 (2.04–2.59)	2.18 (1.93–2.46)	1.90 (1.25–2.89)	1.60 (1.00–2.56)	1.51 (0.94–2.42)
≥ 35	2.01 (1.92–2.11)	1.95 (1.85–2.05)	1.64 (1.56–1.73)	3.18 (2.84–3.55)	2.54 (2.24–2.87)	2.32 (2.05–2.63)	2.64 (1.74–4.01)	2.16 (1.34–3.46)	1.97 (1.23–3.16)
Year of delivery									
1999–2004	1	1	1	1	1	1	1	1	1
2005–2011	0.88 (0.86–0.90)	0.89 (0.87–0.91)	0.88 (0.86–0.90)	0.88 (0.84–0.91)	0.85 (0.82–0.89)	0.85 (0.81–0.89)	0.77 (0.68–0.86)	0.79 (0.70–0.89)	0.80 (0.71–0.90)
Education									
Elementary: 9 ≤ years		0.83 (0.78–0.87)	0.84 (0.79–0.89)		0.92 (0.83–1.02)	0.92 (0.84–1.02)		1.02 (0.83–1.26)	1.01 (0.82–1.25)
High school		1	1		1	1		1	1
College or university		1.08 (1.05–1.12)	1.10 (1.07–1.13)		1.01 (0.96–1.06)	1.03 (0.98–1.08)		0.97 (0.85–1.11)	0.97 (0.85–1.11)
Country/Region of birth									
Sweden		1	1		1	1		1	1
Asia		1.66 (1.58–1.74)	1.58 (1.50–1.65)		1.40 (1.28–1.54)	1.36 (1.24–1.49)		1.43 (1.15–1.78)	1.40 (1.13–1.74)
Africa		2.01 (1.85–2.20)	1.96 (1.79–2.14)		1.76 (1.50–2.06)	1.71 (1.45–2.00)		2.14 (1.58–2.88)	2.05 (1.52–2.77)
Other		0.93 (0.89–0.97)	0.93 (0.88–0.97)		0.95 (0.87–1.03)	0.95 (0.87–1.04)		0.92 (0.73–1.17)	0.94 (0.74–1.18)
Smoking in early pregnancy									
No		1	1		1	1		1	1
Yes		0.81 (0.76–0.86)	0.81 (0.76–0.86)		0.88 (0.78–0.98)	0.87 (0.77–0.97)		0.96 (0.75–1.22)	0.93 (0.73–1.18)
BMI									
Low: <18.5		1.23 (1.14–1.33)	1.22 (1.13–1.31)		1.28 (1.08–1.52)	1.28 (1.08–1.51)		0.71 (0.35–1.43)	0.71 (0.35–1.44)
Normal weight: 18.5–24.9		1	1		1	1		1	1
Overweight: 25–29.9		0.96 (0.93–0.99)	0.95 (0.92–0.99)		1.00 (0.94–1.05)	0.99 (0.94–1.05)		0.92 (0.80–1.06)	0.92 (0.80–1.06)
Obese: ≥ 30		0.90 (0.85–0.94)	0.90 (0.86–0.95)		0.96 (0.89–1.03)	0.96 (0.89–1.04)		0.79 (0.65–0.97)	0.79 (0.64–0.97)
Infant birthweight (g)									
< 3000		1	1		1	1		1	1
3000–3499		1.70 (1.61–1.81)	1.69 (1.60–1.80)		1.45 (1.25–1.67)	1.44 (1.25–1.67)		1.02 (0.68–1.53)	1.02 (0.68–1.54)
3500–3999		2.98 (2.81–3.16)	2.88 (2.72–3.05)		2.53 (2.20–2.91)	2.50 (2.17–2.87)		2.40 (1.64–3.51)	2.38 (1.62–3.48)
4000–4499		5.08 (4.78–5.40)	4.69 (4.41–4.98)		4.25 (3.69–4.90)	4.09 (3.55–4.72)		3.97 (2.70–5.83)	3.84 (2.61–5.65)
≥ 4500		8.51 (7.86–9.20)	7.62 (7.04–8.25)		7.39 (6.34–8.61)	7.01 (6.01–8.17)		8.17 (5.48–12.16)	7.70 (5.16–11.49)
Presentation									
Occiput anterior		1	1		1	1		1	1
Occiput posterior		1.89 (1.78–2.00)	1.43 (1.35–1.51)		1.92 (1.74–2.12)	1.59 (1.44–1.76)		2.00 (1.55–2.59)	1.58 (1.22–2.06)
Breech		0.87 (0.76–0.99)	1.06 (0.93–1.22)		1.20 (0.72–2.02)	1.26 (0.75–2.12)		1.34 (0.33–5.40)	1.34 (0.33–5.42)
Other		1.11 (1.04–1.18)	1.03 (0.97–1.11)		1.24 (1.09–1.41)	1.22 (1.07–1.38)		1.72 (1.30–2.28)	1.70 (1.29–2.25)
History of OASI in 1st pregnancy^3^									
No					1	1			
Yes					4.38 (4.08–4.70)	4.38 (4.09–4.70)			
History of cesarean section 1st pregnancy^3^									
No					1	1			
Yes					7.14 (6.77–7.53)	5.46 (5.14–5.80)			
History of OASI in 2nd pregnancy^4^									
No								1	1
Yes								8.92 (7.28–10.90)	8.62 (7.01–10.61)
History of cesarean section 2nd pregnancy^4^									
No								1	1
Yes								3.29 (2.45–4.41)	2.93 (2.18–3.95)
Diabetes									
No			1			1			1
Yes			0.98 (0.79–1.21)			1.35 (1.04–1.73)			0.98 (0.48–2.02)
Epidural analgesia									
No			1			1			1
Yes			0.93 (0.90–0.95)			1.09 (1.03–1.15)			1.10 (0.93–1.29)
Episiotomy									
No			1			1			1
Yes			0.89 (0.87–0.92)			1.04 (0.96–1.13)			1.34 (1.01–1.77)
Vacuum extraction or forceps									
No			1			1			1
Yes			2.87 (2.80–2.95)			2.82 (2.63–3.02)			4.07 (3.33–4.99)

Adjusted Odds Ratio (OR) with 95% Confidence Interval (CI) in three different models: all variables included in the respective model presented

^1^Multiple imputation of missing values in BMI

^2^Multiple imputation of missing values in BMI, OASI in previous birth, and cesarean section in previous birth

^3^Only second births

^4^Only third births

The following factors included in Model 3 were not statistically significant in any of the three parity groups, and are not shown in the table: pregestational hypertension, gestational diabetes and preeclampsia

When the mediating factors (epidural analgesia, episiotomy, instrumental delivery) and maternal age-related morbidity (diabetes, pregestational hypertension, gestational diabetes, preeclampsia) were added in Model 3, only minor reductions of the risk estimates were found, and these did not alter the general findings.

Table 2 also shows the independent effect of other risk factors for OASI, the strongest of which were infant birth weight of ≥4000 g, history of OASI, and history of cesarean section. Factors that increased the risk in a similar way in all three parity groups were, besides infant birth weight, occiput posterior presentation, African and Asian region of birth, and instrumental vaginal delivery.

## Discussion

This study showed that the risk of OASI in live singleton vaginal births at term increased by maternal age in first, second and third births. The association between increasing maternal age and OASI is supported by studies in which the age distribution is divided into four categories or more [4, 12–14, 16, 26], whereas only two age categories may have concealed the effect [15, 19, 25]. The rates of OASI differed between first, second and third births, indicating that information about parity is important, not only when comparing incidences of OASI in different settings, but also when discussing the effect of maternal age in individual cases. Even though the aged-related risk of OASI was approximately doubled at age 30 and older in both first and second births, compared with the respective reference group aged <25 years, the proportion of women who experienced an OASI was greater in first births due to the higher prevalence at baseline (4.1% and 1.0% at age < 25 years in first and second births respectively, Table 1).

We further explored the finding that risk of OASI at age 35 and older in first births did not continue to increase but was the same as in the previous age category (Fig. 1). Several other studies have reported that the risk of OASI in first births did not increase in the oldest women, mostly defined as 40 years and older [4, 14, 18, 26]. We speculate that one explanation could be that the oldest nulliparae who had a vaginal birth were more low-risk, compared with the oldest women having their second or third vaginal birth, because many having a first birth in this age category were delivered by cesarean section (CS rates at age ≥ 35 years were 30% in 1st births, 21% in 2nd births, and 17% in 3rd births). Infant birthweight of ≥4000 g was a strong risk factor for OASI, and this factor was less prevalent in the oldest nulliparae with a vaginal birth compared with those delivered by CS (birth weight ≥ 4000 g in vaginal birth versus CS at age ≥ 35: 1st births 14% vs. 23%; 2nd births 24% vs. 24%; 3rd births 27% vs. 20%). Although analyses were adjusted for infant birthweight, this outcome could have been associated with other unmeasured confounding, such as prolonged labour.

In third births, the age-related risk of OASI commenced at higher age than in first and second births, and we cannot exclude that this finding was also related to unmeasured confounding. The reference group, with which all other age groups were compared, included only 3.7% of third births, and constituted a very selected group of women with high rates of low education, non-Swedish background, smoking and high BMI. This selection of women may be explained by the fact that having a third child before the age of 25 years is uncommon in Sweden where two children has long been the norm [33, 34]. However, since the end of the 1990s it has become more popular to have a third child, and this trend has primarily involved highly educated women and high income earners [35]. Consequently, third births included a more heterogeneous group of women in terms of socioeconomic background and lifestyle, both factors associated with OASI.

The physiological mechanism explaining why rates of OASI are much lower in multiparous than primiparous women may primarily be related to more resilient anatomical structures, after having been stretched during the first birth. The selection of women who have more than one child may also be a consideration, since OASI in the first birth may be a reason for not having more children. This makes multiparous women more low risk in relation to OASI, which was illustrated in the present study with a history of OASI being least common in third births.

The physiological mechanisms explaining the independent effect of maternal age on the risk of OASI seem more complex. Although we know that ageing occurs in all of the body’s cells, tissues and organs from an early age, the physiological effect of ageing on the connective and muscular tissues in the perineal region during childbirth is poorly researched. One effect of ageing which could possibly affect blood circulation in the perineum is the early onset of sclerotic lesions [36]. Of all the measures taken to reduce rates of OASI, such as manual support to reduce pressure on the perineum [37, 38], injection of hyaluronidase to affect perineal connective tissue [39, 40], application of oil [41] and antenatal massage [42, 43] to increase elasticity in the perineum, only application of warm packs to increase blood circulation in the perineum [44] has been effective. Whether perineal warm packs are more effective at advanced age has not, however, been investigated. Our finding that smoking decreased the risk of OASI was confusing, considering that smoking has been described as an ageing accelerator, which exhausts cellular defense and repair functions [45]. Norwegian [8] and Finnish [18] cohort studies have also reported a lower risk of OASI in smokers, and Räisänen and colleagues suggested that “smoking may interfere with collagen synthesis and connective tissue properties, and thus modify the OASI risk by an, as yet, unknown mechanism.” (page7).

This study shows that giving birth for the first time at a young age could reduce the risk of OASI, with maternal age under 25 years being most optimal. However, to change the current trend in modern societies of parenthood often starting after the age of 30, is probably not the most effective or feasible way of achieving a reduction in the incidence of OASI at a population level. The most important step is to decrease the adverse effects of OASI by effective diagnosis, followed by effective treatment. Faltin and colleagues [46] showed in a randomized controlled trial of primiparous women without a clinically evident anal sphincter tear that endoanal ultrasound examination of the perineum after childbirth increased the detection rate of OASI to 5.6%, and that immediate repair decreased the risk of severe fecal incontinence 3 months after birth from 8.7% in the control group to 3.3% in the intervention group. A systematic review of seven observational studies reported a reduction of OASI after the introduction of a manual support technique originating from Finland [24]. The level of evidence in the included studies was, however, too low to recommend implementation in clinical settings.

The strengths of this study include the high quality of data in the Swedish Medical Birth Register, with a low percentage of missing cases (<2%), and the possibility of taking important confounding factors into account. Including history of OASI and cesarean section in the analyses of second and third births was particularly important considering the strong associations between these variables and OASI. Still, residual confounding may be a problem, and information was not available on important confounders, such as length of second stage labour, inter-delivery interval and female genital mutilation. Another limitation was that the proportion of missing values was relatively high in relation to BMI and history of OASI and cesarean section. The missing values in these variables were assumed to be close to random which justified multiple imputation. The findings presented in this study differed little from those obtained by complete case analyses, and none of the conclusions was altered.

## Conclusions

Our study suggests that advanced maternal age is an independent risk factor for OASI irrespective of parity. When discussing young people’s life plans, this information could be added to other information about the potential risks associated with postponing parenthood to age 30 and later. For the clinician who assists women during labour, increased attention should be paid to older women, especially nulliparae, in order to ascertain the status of the perineum and optimise prompt treatment when OASI is diagnosed.

## Additional file

Additional file 1: Table S1 Risk of OASI in first, second and third vaginal births at term with a live singleton fetus. Comparisons between complete case analyses and analyses based on imputed data as in Table 2 Model 2. (XLSX 16 kb)
